# The IL-8 protease SpyCEP is detrimental for Group A *Streptococcus* host-cells interaction and biofilm formation

**DOI:** 10.3389/fmicb.2014.00339

**Published:** 2014-07-10

**Authors:** Federica Andreoni, Taiji Ogawa, Mariko Ogawa, Jerzy Madon, Satoshi Uchiyama, Reto A. Schuepbach, Annelies S. Zinkernagel

**Affiliations:** ^1^Division of Infectious Diseases and Hospital Epidemiology, University Hospital Zurich, University of ZurichZurich, Switzerland; ^2^Surgical Intensive Care Medicine, University Hospital Zurich, University of ZurichZurich, Switzerland

**Keywords:** Group A *Streptococcus*, SpyCEP, IL-8 protease, colonization, biofilms, virulence factors

## Abstract

SpyCEP-mediated chemokine degradation translates into more efficient spreading and increased severity of invasive Group A *Streptococcus* (GAS) infections, due to impaired neutrophil recruitment to the site of infection. SpyCEP is markedly up-regulated in invasive as compared to colonizing GAS isolates raising the question whether SpyCEP expression hinders bacterial attachment and thus colonization of the host. To address this question we used a molecular approach involving the use of homologous GAS strains either expressing or not SpyCEP or expressing an enzymatically inactive variant of SpyCEP. We found that expression of enzymatically functional SpyCEP lowered GAS adherence and invasion potential toward various epithelial and endothelial cells. SpyCEP also blunted biofilm formation capacity. Our data indicate that expression of SpyCEP decreases colonization and thus might be detrimental for the spreading of GAS.

## Introduction

Group A *Streptococcus* (GAS or *Streptococcus pyogenes*), a Gram positive bacterium, is a strict human pathogen causing a large burden of disease worldwide. GAS diseases range from mild to severe and life-threatening, resulting in an estimate of 517,000 deaths each year (Carapetis et al., 2005). GAS expresses a vast range of virulence factors that are involved in blunting and evading the host immune system favoring bacterial spreading. Up-regulation of virulence factors is found in invasive GAS isolates and distinguishes them from most colonizing strains (Sumby et al., 2006). When transitioning to invasive strains a distinct phenotype is found in about 40% of clinical disease-causing isolates. The phenotype is characterized by the up-regulated virulence factors Streptolysin O (SLO), extracellular DNase (Sda1) and *Streptococcus pyogenes* cell envelope protease (SpyCEP) promoting the spreading of the infection by interfering with recruitment and function of phagocytic cells (Sumby et al., 2006).

One of the most highly up-regulated virulence factors in invasive disease is SpyCEP (Sumby et al., 2006). SpyCEP (aka Spy0416, ScpC, CepA, Prts, or SP24) has the ability to cleave human CXC-chemokines such as GCP-2 (granulocyte chemotactic peptide-2, CXCL6), GRO-α, β, γ (growth related oncogene-α, CXCL1, 2, 3), ENA-78 (neutrophil-activating peptide-78, CXCL5), Grap-2 (GRB2-related adapter protein 2) and IL-8 (CXCL8) which correspond to the murine CXC-chemokines MIP-2 and KC (Hidalgo-Grass et al., 2004; Sumby et al., 2006; Chiappini et al., 2012). The presence of a bacterial chemokine-degrading enzyme was first described in a patient suffering from necrotizing fasciitis. Histology revealed a striking paucity of neutrophils despite the massive streptococcal infection and was found to be due to IL-8 degradation by GAS (Hidalgo-Grass et al., 2004). The protein responsible for IL-8 degradation was later identified as SpyCEP, a subtilisin-like serine protease present in the supernatant of various GAS blood isolates (Edwards et al., 2005). The expression of SpyCEP is regulated by SilCR and CovR/S (Hidalgo-Grass et al., 2004; Sumby et al., 2008). The protease contains the LPXTG cell wall-anchoring motif (Sumby et al., 2008) present on the cell wall during bacterial log phase growth and shed into the supernatants of bacterial cultures during stationary phase growth (Chiappini et al., 2012). In its mature form SpyCEP is present as a dimer composed of 2 subunits (30 and 150 kDa) generated by intramolecular autocatalytic cleavage. The 30 kDa subunit contains one of the 3 amino acids composing the active site and a stable interaction between the 2 subunits is necessary for enzymatic activity (Zingaretti et al., 2010).

We previously showed that SpyCEP impairs neutrophil endothelial transmigration, promotes resistance to neutrophil killing by reducing the production of Neutrophil Extracellular Traps (NETs) and decreases adherence to and invasion of epithelial Hep-2 cells (Zinkernagel et al., 2008). In *in vivo* mouse models of invasive and necrotic skin infection, GAS strains expressing SpyCEP caused more severe infections as revealed by the presence of larger skin lesions and increased mortality (Hidalgo-Grass et al., 2006; Zinkernagel et al., 2008). These murine *in vivo* results are corroborated by clinical findings that invasive clinical GAS isolates display higher SpyCEP activity than the superficial ones (Edwards et al., 2005). However, using GAS WT strains other than the M1T1 strain as well as different mouse and infection models, SpyCEP-deficient mutants were also found to cause larger skin lesions as compared to the SpyCEP-expressing strain (Sjolinder et al., 2008; Sumby et al., 2008). Possibly such divergent findings could be explained by extensive and therefore harmful neutrophil recruitment to sites of infection in the absence of SpyCEP and thus causing larger local tissue damage. In an artificial system based on polystyrene beads coated with recombinant SpyCEP, SpyCEP was found to promote uptake of the beads by HUVECs cells (Kaur et al., 2010). No attachment or internalization was however observed for the epithelial cell lines Hep-2 and A549 and SpyCEP did not mediate invasion when heterologously expressed in Group B *streptococci* (Kaur et al., 2010). This underlines the fact that an artificial system constituted by beads coated with a single protein can act very differently from live bacteria where many more factors present on the cell surface come into play. Up-regulation of most GAS virulence factors has been shown to boost GAS pathogenicity at the time of invasion. However, previous findings describe up-regulation of SpyCEP as detrimental for the colonization of tissue (Zinkernagel et al., 2008). We thus investigated whether the expression of SpyCEP in GAS WT or the lack, thereof using isogenic SpyCEP knockout strains, affected adherence to and invasion of endothelial and epithelial cells. We found that the presence of a functional SpyCEP was detrimental for adherence as well as invasion and that the absence of SpyCEP significantly facilitated biofilm formation.

## Materials and methods

### Cell lines

The epithelial cell lines Detroit-562 (human pharyngeal cancer cells) (Peterson et al., 1968) and A549 (adenocarcinomic human alveolar basal epithelial cells) and the primary endothelial cells HUVEC (human umbilical vein endothelial cells, Roche Clonetics) as well as the endothelial cell line Ea.hy926 (immortalized human umbilical vein cells) (Edgell et al., 1983) were used in this work.

### Group a *streptococcus* (GAS) strains

The wild type GAS M1T1 strain 5448 (GAS WT) (Chatellier et al., 2000) was used together with its derivatives GAS Δ*cepA*, devoid of SpyCEP expression (Zinkernagel et al., 2008), GAS Δ*cepA* compl, in which SpyCEP expression was restored by complementation with the pDesterm*cepA* plasmid (Zinkernagel et al., 2008) and GAS Δ*cepA* compl^*^, expressing an inactive form of SpyCEP (SpyCEP^*^). GAS Δ*cepA* compl^*^ was constructed by transformation of GAS Δ*cepA* with the pDestermcepA^*^ plasmid containing a mutated *cepA* gene (cepA^*^). pDestermcepA^*^ was created by introducing a point mutation exchanging the aspartic acid in position 151 with an alanine in the *cepA* gene contained in pDesterm*cepA* (Zingaretti et al., 2010). The QuickChange XL Site-Directed Mutagenesis kit (Stratagene) was used to introduce the point mutation using the primers: *cepA*_D151A_fwd GTTGTCGCAGTTATTGCCACAGGGATCGATCCG and *cepA*_D151A_rev CGGATCGATCCCTGTGGCAATAACTGCGACAAC. The mutation was verified by sequencing and absence of SpyCEP activity was documented using an IL-8 degradation assay, as previously described (Zinkernagel et al., 2008).

### Growth media

A549 and Ea.hy926 cells were grown in Dulbecco's Modified Eagle's Medium (DMEM, Gibco) supplemented with L-glutamine, Detroit-562 cells were grown in Eagle's minimal essential medium (EMEM, Sigma) and HUVEC cells were grown in Endothelial cell growth medium-2 (EBM-2, Lonza), according to the manufacturer specifications. 10% fetal calf serum (FBS, PAA) was added to all culture media. GAS strains were grown in Todd–Hewitt broth (THB, BD) medium supplemented with 2% yeast extract (THY, Oxoid) or on THY-agar plates. Strains bearing the pDCerm-derived plasmids were grown in the presence of 3 μg/ml erythromycin (erm). Phosphate–buffered saline (PBS) was used for washing.

### Adherence and invasion assays

Adherence and invasion assays were carried out as previously described (Uchiyama et al., 2013). Shortly, epithelial and endothelial cells were seeded in 24 well plates. GAS strains were grown O/N in THY medium, diluted 1:10 on the day of the experiment and grown to an OD600 value of 0.4, at which point they were diluted in cell growth medium +0.4% bovine serum albumin (Sigma) and added to the cells at an MOI of 1 or 10 for adherence and invasion, respectively. The cells were then incubated for 30 min (adherence) or 2 h (invasion) at 37°C and 5% CO_2_. For adherence assays the cells were washed 6X in PBS, trypsinized and lysed with 0.02% triton-X100. Serial dilutions of the lysates were plated on THY-agar plates and bacterial units enumerated after O/N incubation at 37°C. For invasion assays, the cells were washed 3X in PBS after 2 h incubation with GAS followed by incubation in the presence of penicillin and gentamycin (10 and 100 μg/ml) for 2 h, washed 3X in PBS and then processed as for the adherence assay.

### Confocal laser scanning microscopy

The human epithelial cells Detroit-562 were seeded in culture medium into a 24-well microtiter plate containing glass coverslips and incubated for 24–48 h. Bacterial cells were stained using carboxyfluorescein-diacetate-succinimidyl ester (CFSE, Invitrogen), and grown to OD_600_ = 0.4. After washing bacteria were added at an MOI of 20. After centrifugation s at 1200 *g* for 5 min, the cells were incubated at 37°C for 30 min. Nuclei were stained with 4',6-diamidino-2-phenylindole (DAPI, Sigma), fixed overnight with 4% paraformaldehyde and mounted with ProlongGold antifade reagent (Life Technologies, Carlsbad, CA, USA). Confocal microscopy images were acquired using Zeiss LSM 510 META (Carl Zeiss Microscopy GmbH, Jena, Germany) and processed with IMARIS ver. 7.6.1 (Bitplane AG, Zurich, Switzerland). The integrated densities of bacteria on the surface of cell monolayers were evaluated using the analysis-measure tool of the ImageJ software 1.47v.

### Biofilm formation assay

Biofilm formation was determined using crystal violet staining of adherent biofilm, as previously described (Manetti et al., 2007; Ogawa et al., 2011). Briefly, overnight cultures were grown in THY medium, diluted 10-fold with DMEM and seeded into 96-well plates. Each bacterial strain was seeded into 6 wells and incubated at 37°C for 24 h. After removal of medium, the plates were washed 3 times with PBS, and adherent bacteria were stained with 0.2% crystal violet for 2 min and washed 3 times with PBS. Stained bacteria were eluted with 100 μl of 100% ethanol and the intensity of crystal violet staining was measured by assessing the absorbance at 595 nm (OD_595_).

### SpyCEP^*^ activity

SpyCEP^*^ activity was measured via Western blot as a function of IL-8 degradation, as published (Zinkernagel et al., 2008). An ELISA-based approach was also undertaken to quantify IL-8 degradation. Briefly, 9 μl of filtered bacterial supernatants from OD_600_ 0.4 GAS cultures were incubated O/N at 37°C with 1 μl of IL-8 (final concentration 0.1 ng/ml) and an ELISA assay (R&D Systems) was subsequently performed.

### SpyCEP-mediated protein cleavage assay

SpyCEP-mediated protein cleavage was carried out using 100 ng/ml of recombinant SpyCEP in PBS. Recombinant SpyCEP (rSpyCEP) was produced in *E. coli* as previously described (Kaur et al., 2010).

### Quantification of hyaluronic acid content

Hyaluronic acid was extracted from bacterial pellets in mid-logarithmic growth phase as described earlier (Hollands et al., 2010). The samples were diluted and the hyaluronic acid was quantified using a commercial test kit (Corgenix) according to the manufacturer's instructions.

### Quantification of endothelial adherence surface proteins

In confluent Ea.hy926 cells surface antigens were determined as previously described (Schuepbach et al., 2008). In brief cells were incubated with either buffer alone, rSpyCEP (10 μg/mL) or the same concentration of a control recombinant protein (rS1LG) isolated using the same technique as rSpyCEP, 2 h before fixation with 2% PFA for 30 min at 4°C. Followed by addition of murine monoclonal antibodies targeting β-1-integrin (β-int), ICAM, VCAM or the endothelial cells integrity markers CD144 and thrombomodulin (TM). A horseradish peroxidase (HRP)–coupled goat anti-mouse antibody and tetrametylbenzidine were used for spectrophotometric quantification of specific cell surface antibody binding.

### Scanning electron microscopy

GAS WT and GAS Δ*cepA* were cultured to mid-log phase (OD_600_ = 0.4) and adjusted with PBS to 2 × 10^9^ CFU/ml, followed by fixation with 2.5% glutaraldehyde for 24 h at 4°C followed by placement on cover glass slide by cytospin at 1000 g for 3 min. Bacterial samples were postfixed with 1% osmium tetroxide followed by dehydration with increasing concentrations of ethanol (70–100%). After critical point drying (Bal Tec CPD 030 Critical point dryer, Leica Biosystems), the samples were coated with platinum using sputter-coating system (Bal Tec SCD 500 Sputter Coater, Leica Biosystems) and then examined with a field emission scanning electron microscopy (Supra 50 VP, Carl Zeiss) using inlens detector.

### Statistics

All statistical analyses were performed using a two-tailed unpaired *t*-test.

## Results

### Enhanced GAS adherence to and invasion of epithelial and endothelial cells in the absence of spyCEP

**Figure 1:**
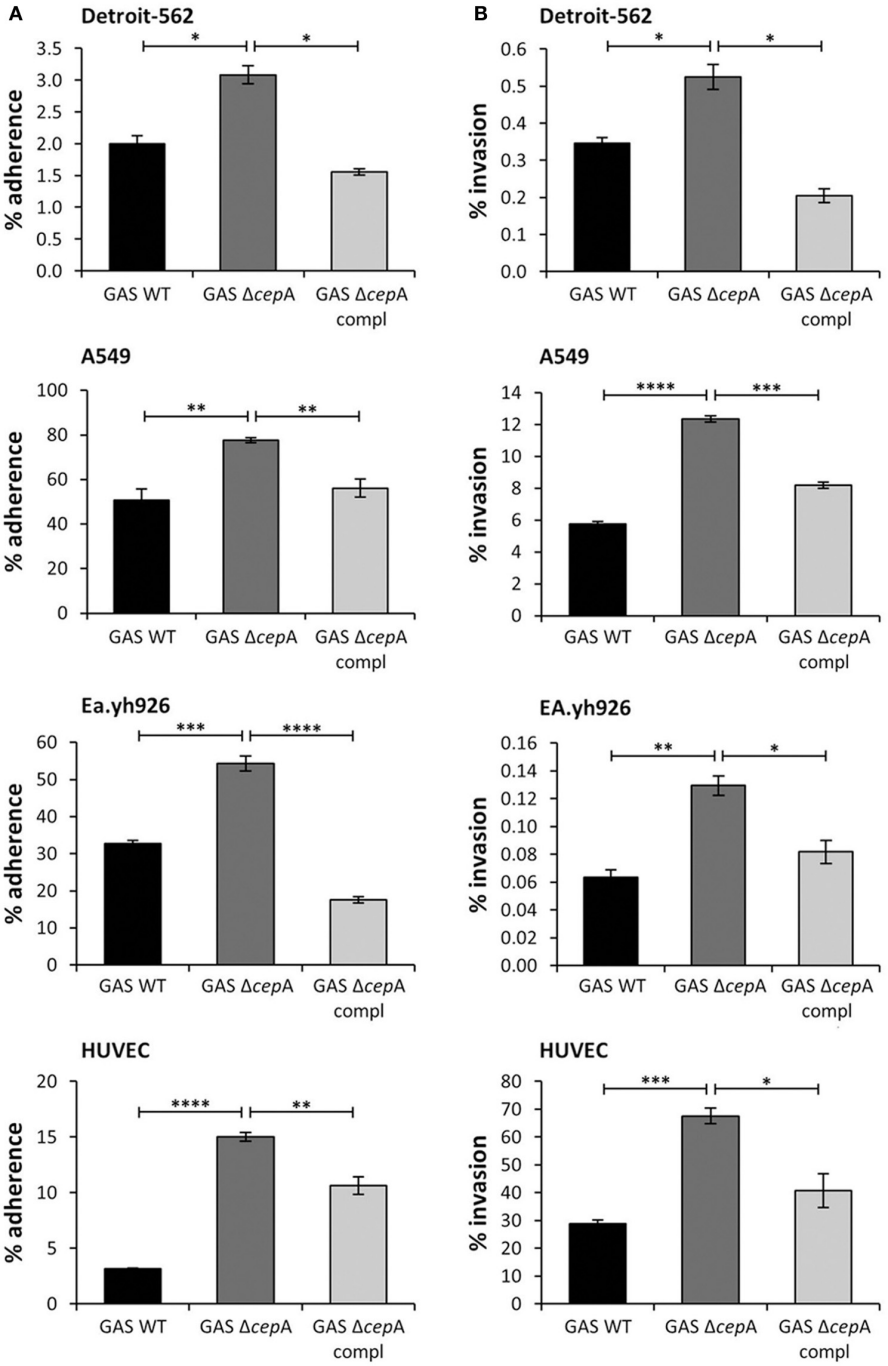
**Enhanced GAS adherence to and invasion of epithelial and endothelial cells in the absence of SpyCEP**. The epithelial cell lines Detroit-562 and A549 and the endothelial cells Ea.hy926 and HUVECs were challenged with the GAS strains GAS WT, GAS Δ*cepA*, and GAS Δ*cepA* compl. MOI of 1 was used for adherence assays and MOI of 10 was used for invasion assays. To assess adherence and invasion an antibiotic protection assay was used and adhering/invading bacteria enumerated by plating and colony counting. **(A)** GAS adherence to and **(B)** invasion of the epithelial and endothelial cells were assessed after 30 min and 2 h respectively. The absence of SpyCEP significantly increased adherence to and invasion of epithelial and endothelial cells by GAS. Each experiment was carried out in triplicate; the figure shows one representative experiment. Error bars represent the standard error, statistical analysis was carried out using a two-tailed *t*-test (ns = *p* > 0.05, ^*^p ≤ 0.05, ^**^p ≤ 0.01, ^***^p ≤ 0.001, ^****^p < 0.0001).

SpyCEP is barely expressed by superficial but largely induced in invasive GAS strains (Sumby et al., 2006). We thus wondered whether the virulence factor SpyCEP might influence the adherence and invasion potential of GAS in epithelial and endothelial cells. In order to test this we used the highly invasive M1T1 strain expressing SpyCEP (Zinkernagel et al., 2008) and found that in its isogenic SpyCEP-deficient counterpart, GAS Δ*cepA*, adherence to and invasion of epithelial as well as endothelial cells was significantly enhanced (Figures 1A,B). Complementation of GAS Δ*cepA* with a plasmid containing the *cepA* gene (GAS Δ*cepA* compl) restored the wild type phenotype resulting in lower adherence and invasion.

### Reduced adherence and invasion potential due to loss in spyCEP activity

**Figure 2:**
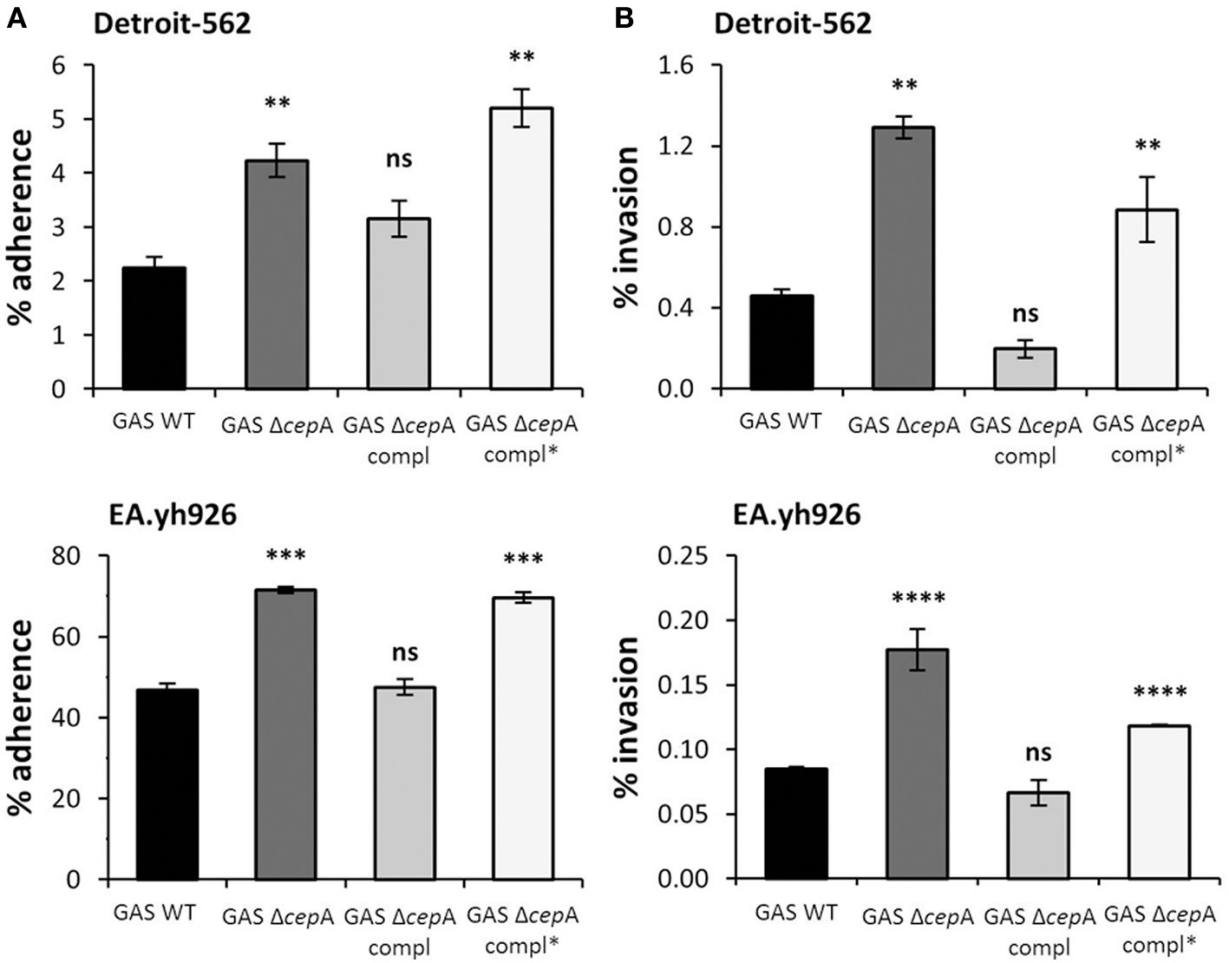
**SpyCEP activity requirement for GAS adherence to and invasion of epithelial and endothelial cells**. Representative epithelial (Detroit-562) and endothelial (EA.hy926) cell lines were challenged with GAS WT, GAS Δ*cepA*, GAS Δ*cepA* compl and GAS Δ*cepA* compl^*^. **(A)** Adherence and **(B)** invasion assays were carried out as described in Figure 1. GAS Δ*cepA* compl^*^ behaved like GAS Δ*cepA* in terms of adherence and invasion to Detroit-562 and EA.hy926 cells. Each experiment was carried out in triplicate and the graphs show one representative experiment. Error bars represent the standard error, statistical analysis was carried out using a two-tailed *t*-test (ns = *p* > 0.05, ^*^p ≤ 0.05, ^**^p ≤ 0.01, ^***^p ≤ 0.001, ^****^p < 0.0001). The *p*-values are relative to GAS WT.

In order to investigate whether SpyCEP activity affected adherence and invasion, GAS Δ*cepA* was transformed with a plasmid containing a mutated version of SpyCEP devoid of serine protease activity (GAS Δ*cepA* compl^*^). SpyCEP^*^ expression was verified by binding studies using anti-SpyCEP antiserum (Figure S1A). Loss of SpyCEP activity in GAS Δ*cepA* compl^*^ was assessed as a measure of IL-8 degradation both via ELISA and Western blot (Figure S1B). The adherence and invasion potential of GAS Δ*cepA* compl^*^ was tested on Detroit-562 cells as representative epithelial cells and on Ea.hy926 cells as representative endothelial cells. Similarly to the GAS Δ*cepA* strain adherence and invasion of GAS Δ*cepA* compl^*^ was found to be significantly higher compared to GAS WT suggesting that SpyCEP activity *per se* impedes GAS adherence and invasion (Figures 2A,B).

### Enhanced attachment and biofilm formation in the absence of spyCEP

**Figure 3:**
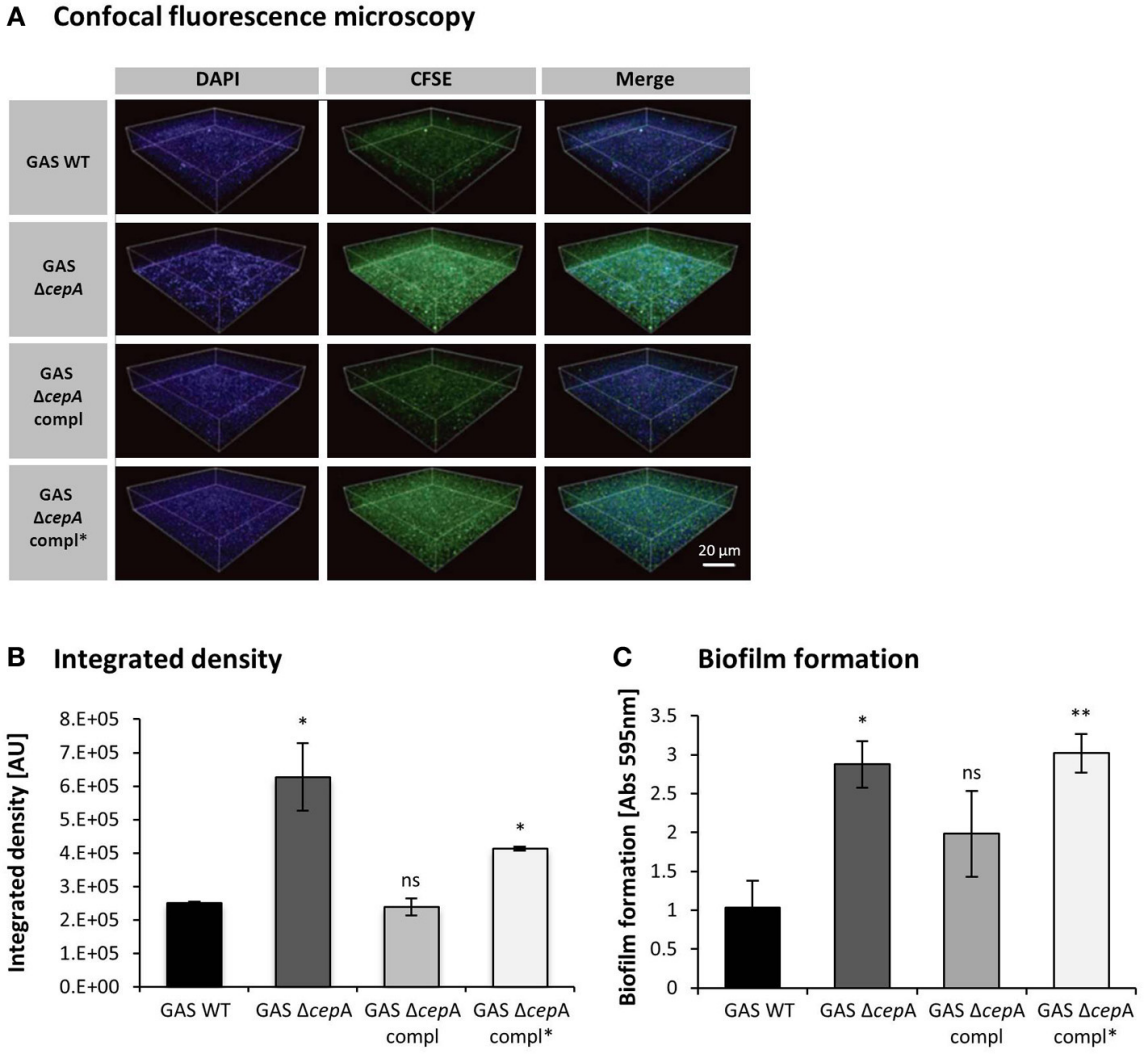
**Decreased GAS cell surface attachment and biofilm formation in the presence of SpyCEP. (A)** Microscopy analysis of GAS attached to the surface of human pharyngeal cells. GAS strains and Detroit-562 cells were stained with CFSE (green) and DAPI (blue) respectively and attachment was assessed using a fluorescence confocal laser scanning microscope. The scale bar in the confocal images represents 20 μm. **(B)** Integrated densities of attached bacteria obtained from confocal images were evaluated using ImageJ. The integrated density is measured in arbitrary units (AU). Data were pooled from three independent experiments done in triplicate, mean ± *SD*; ^*^p < 0.01, compared to GAS WT. **(C)** Biofilm formation was assessed for GAS WT, GAS Δ*cepA*, GAS Δ*cepA* compl and GAS Δ*cepA* compl^*^. The absence of SpyCEP or the presence of an inactive SpyCEP form resulted in increased biofilm formation. The graph represents pooled data from three independent experiments; the error bars represent the standard deviation. Statistical analysis was carried out using a two-tailed *t*-test (ns = *p* > 0.05, ^*^p ≤ 0.05, ^**^p ≤ 0.01). The *p*-values are relative to GAS WT.

The attachment of the GAS strains to Detroit-562 cells was quantified by confocal fluorescence microscopy showing higher attachment in the absence of SpyCEP protein or in the presence of an inactive SpyCEP version (Figures 3A,B). Enhanced cellular invasion, efficient colonization and persistence of bacteria have been linked to biofilm-forming capacity (Sekhar et al., 2009; Ogawa et al., 2011). We thus investigated in a next step whether the absence of SpyCEP would influence biofilm formation and thereby alter GAS colonization potential. We found biofilm formation to increase when SpyCEP was not present or non-functional (Figure 3C).

### SpyCEP activity does not influence adhesion molecules structure and expression

**Figure 4:**
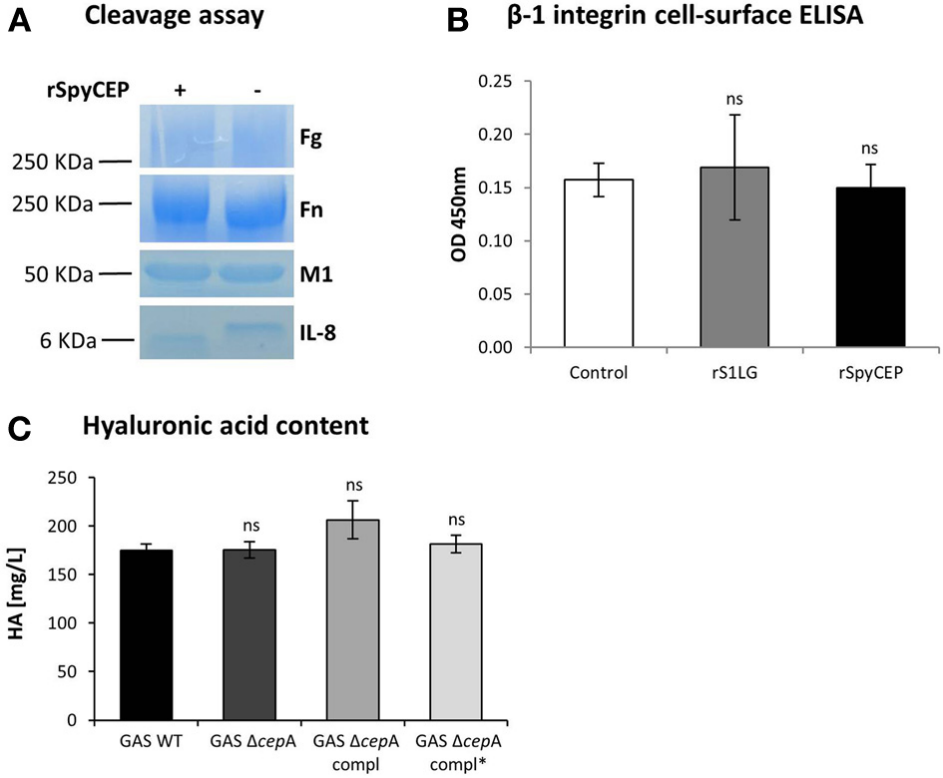
**Assessment of SpyCEP cleavage and hyaluronic acid content. (A)** SpyCEP-mediated cleavage of proteins involved in bacteria-host cells interactions was assessed. After overnight incubation in the presence of recombinant SpyCEP (rSpyCEP, 100 ng/ml), the samples were loaded on a polyacrylamide gel and cleavage assessed by coomassie blue staining. IL-8 was used as a positive control for the cleavage reaction. **(B)** β-1 integrin expression on EA.hy926 cells surface after exposure to buffer only (control), rSpyCEP (10 μg/ml) or an unrelated protein (S1LG, 10 μg/ml) purified using the same method. The graph represents two pooled experiments and error bars represent the standard deviation. **(C)** Hyaluronic acid (HA) content quantification in the GAS strains. Error bars represent the standard deviation, statistical analysis was carried out using a two-tailed *t*-test (ns = *p* > 0.05). The *p*-values are relative to GAS WT.

To exclude that SpyCEP hindered bacterial adherence by cleavage of fibrinogen, fibronectin, or the M1 protein, among the main factors involved in GAS adherence to host cells, we performed cleavage assays. We found that none of these host or bacterial proteins was cleaved by rSpyCEP (Figure 4A). To reveal other possible mechanisms by which SpyCEP activity could lead to reduced adherence and invasion of host cells, a cell-surface ELISA targeting the host adhesion molecule β-1 integrin was performed on the endothelial cell line Ea.hy926 in the presence or absence of rSpyCEP or an unrelated protein (rS1LG) purified using the same method. β-1 integrin can interact with fibronectin and therefore mediate attachment of GAS (Ruoslahti, 1991). No difference in the β-1 integrin amount present on the host cells surface upon exposure to rSpyCEP was observed (Figure 4B). The same result was obtained for the adhesion molecules VCAM and ICAM and for the endothelial cells integrity markers VE-cadherin (CD144) and thrombomodulin (TM) (Figure S2A). Scanning electron microscopy showed similar surface quality (Figure S3). Hyaluronic acid capsule content influences adherence (Cho and Caparon, 2005; Hollands et al., 2010). Thus we quantified the hyaluronic acid content of the various GAS strains and again found no difference (Figure 4C). It can therefore be concluded that SpyCEP-mediated modification of the aforementioned factors is not responsible for variations in the colonization potential of GAS Δ*cepA*.

## Discussion

We show that expression of functional SpyCEP is detrimental for adherence to and invasion of human epithelial and endothelial cells as well as for biofilm formation, underscoring the importance of virulence factors regulation for bacterial pathogenesis. Our findings in GAS M1T1 support that SpyCEP interferes with host cell adherence and invasion and thus impedes GAS colonization. Our data is consistent with epidemiologic studies reporting that superficial GAS strains in general barely express SpyCEP (Sumby et al., 2006). In colonization and superficial infections bacterial virulence factors are usually not highly expressed which, according to the trade-off hypothesis, allows the bacterium to multiply and persist within the host without causing too much damage allowing perpetuation of the species (Wollein Waldetoft and Raberg, 2014). Evolutionary speaking, the expression of virulence factors triggered in severe invasive diseases should therefore be an obstacle to the maintenance and spreading of the colonizing GAS population. Thus GAS has evolved a strategy to maintain the expression of virulence factors while colonizing and to allow their expression only under particular conditions, e.g., invasive outbursts. The two component regulatory system *covRS* allows down-regulation of GAS virulence factors ensuring a balance between bacterial spreading and host survival. During invasive infections however, the expression of virulence factors is essential for the survival of GAS; from here their maintenance in the genome and the possibility to up-regulate their expression level according to the situation.

Previous reports found that upon invasion SpyCEP is among the most strongly up-regulated virulence factors in GAS due to a point mutation in *covRS* resulting in de-repression of virulence genes (Sumby et al., 2006). Consistently, SpyCEP has been highlighted for boosting virulence in invasive disease through IL-8 degradation thereby inhibiting neutrophil migration and bacterial killing (Zinkernagel et al., 2008). Inactivation of the *covRS* virulence regulator results in higher expression of various bacterial virulence factors, including SpyCEP, without affecting fitness cost in invasive infections but hindering colonization (Alam et al., 2013). In accordance, in a murine naso-pahryngeal infection model, inactivation of the *covRS* virulence regulator was reported to impair shedding and transmission of bacteria by the mice (Alam et al., 2013). Also, a shorter naso-pharingeal carriage was shown for bacteria possessing a functional *rocA* gene able to increase *covR* transcription and therefore able to enhance *covRS*-regulated virulence factors transcription, including SpyCEP (Lynskey et al., 2013). All these findings suggest that the presence of SpyCEP might lead to a loss in colonization potential.

We show that SpyCEP indeed interferes with GAS colonization of epithelial cell lines, representing the lining of the major sites of GAS colonization in humans such as the oropharynx and the lungs (Aronoff and Mulla, 2008). We used a molecular approach to test whether SpyCEP expression was required for interference with colonization of eukaryotic cells. Consistent with our conclusion the isogenic GAS Δ*cepA* strain, deficient in SpyCEP expression and thus comparable to a colonizing strain, possessed a much higher adherence and invasion potential than its GAS WT counterpart. Complementation of GAS Δ*cepA* with a plasmid containing the SpyCEP gene restored the phenotype of the invasive GAS WT strain further supporting our conclusion that SpyCEP affects the ability of GAS to colonize both epithelial and endothelial cells.

Biofilm formation promotes bacterial survival and persistence on living and non-living surfaces. We found that GAS WT attached less to Detroit-562 cell layers and formed less biofilms compared to its isogenic SpyCEP mutant. Complementation of GAS Δ*cepA* to restore SpyCEP expression again restored the wild type phenotype. To our knowledge this is the first report highlighting the involvement of SpyCEP in modulating biofilm formation.

To better understand whether SpyCEP enzymatic activity *per se* influences host cells colonization we examined its role in colonization. SpyCEP is a serine protease and thus it seemed likely that the protease activity would be causative. To test whether enzymatic or structural properties of SpyCEP interfere with GAS colonization we engineered the active site mutant SpyCEP^*^ as published by Zingaretti et al. (2010). We found that heterologous expression of SpyCEP^*^ resulted in the same phenotype as lack of SpyCEP expression whereas heterologous expression of wild type SpyCEP restored the wild type phenotype pointing toward SpyCEP activity as a possible cause for impaired colonization. As previously described, the active form of SpyCEP undergoes maturation through autocatalytic self-cleavage that gives rise to two fragments of 30 and 150 kDa containing one and two of the amino acids composing the catalytic triad of the enzyme's active site respectively (Zingaretti et al., 2010). SpyCEP activity depends on the correct assembly of these two fragments which leads to the formation of an active enzyme. Although the secondary structure of SpyCEP^*^ was reported to be very similar to the one of wild type SpyCEP (Zingaretti et al., 2010), correct assembly of the two subunits will not occur in GAS Δ*cepA* compl^*^ as a result of lack of SpyCEP auto-cleavage. Based on these considerations we hypothesized that loss of enzyme function results in miss-assembly of SpyCEP^*^ resulting in turn in a structure that either influences bacteria-host cell interactions or disables cleavage of factors important for GAS adherence, invasion and biofilm formation.

To determine whether the absence of SpyCEP activity or the absence of a correctly folded enzyme would be causative for the observed increase in colonization potential, we set up a series of cleavage assays involving SpyCEP and some of the main factors involved in GAS adherence to host cells. Potential target substrates of SpyCEP activity are bacterial virulence factors and host cell receptors (Ender et al., 2013) or bacterial binding targets. Among the main bacterial factors known to modulate GAS ability to colonize the host, we found that SpyCEP did not cleave the M1 protein or affect hyaluronic acid content. M1 was shown to be the major factor present on the GAS surface mediating binding to host cells through interaction with fibrinogen (Uchiyama et al., 2013). A very high content in hyaluronic acid capsule is deemed responsible for decreased bacterial attachment to host cells possibly due to masking of bacterial surface adhesins (Hollands et al., 2010) although the effect seems to be serotype-dependent as encapsulation was shown not to be detrimental for pharyngeal infection for a emm18 GAS strain (Lynskey et al., 2013). On the host's side we found that neither fibrinogen nor fibronectin, two of the host proteins known to mediate adherence, were cleaved by SpyCEP. The amount of β-1 integrin found on the host cell surface was also not affected by SpyCEP activity clearly indicating that the disruption of the fibonectin-mediated interaction between GAS and host cells is not the cause of loss in cell attachment. Indirect effects of SpyCEP via cleavage of IL-8 seemed unlikely, since the addition of exogenous IL-8 did not influence adherence and invasion in our hands (data not shown). Previously we also showed that the family of mammalian protease sensing receptors, the protease-activated receptors PAR, was not activated by SpyCEP, in contrast to other GAS proteases, excluding also this pathway from the possible mechanisms mediating modulation of GAS colonization abilities (Ender et al., 2013).

GAS interaction with host cells is mediated by a vast variety of molecules, called adhesins, present on the cell surface that interact with various types of receptors present on the host cells (for an extensive review see Nobbs et al., 2009). Although we screened a few of these factors to find possible ways by which SpyCEP could interfere with host cells adherence and invasion, a comprehensive screening would prove very challenging. The first step involved in the adherence of GAS to host cells is mediated by electrostatic interactions between bacterial and host cell surfaces followed by the establishment of stronger bonds between adhesins and host cell receptors (Hasty et al., 1992). Changes in the electrostatic charge on the bacterial surface could therefore interfere with GAS adherence to host cells. The presence of the active form of SpyCEP on the GAS surface could be acting by altering electrostatic charges therefore undermining the establishment of the first contact with the host cells. Another way by which the protease activity of SpyCEP might interfere with adherence is by hindering the action of bacterial sortases, i.e., proteins that mediate the exposure of adhesins to the bacterial cell surface allowing for interactions with host cells to happen (Marraffini et al., 2006). Our data on adherence, invasion and biofilm formation strongly suggest that the expression of functional SpyCEP is detrimental for promoting colonization of tissues and, in the long run, detrimental for the persistence and subsequent spreading of a superficial infection. Taken together our work illustrates the importance of bacterial virulence factor regulation and how this influences disease. The GAS virulence factor SpyCEP is down-regulated during colonization since it is detrimental for colonization but up-regulated upon invasion where it boosts virulence in invasive disease.

### Conflict of interest statement

The authors declare that the research was conducted in the absence of any commercial or financial relationships that could be construed as a potential conflict of interest.

## Supplementary material

Figure S1 **Expression and activity of SpyCEP^*^. (A)** FACS analysis of anti-SpyCEP antiserum binding to various GAS strains' surface. **(B)** Quantification of SpyCEP activity in the supernatants of the various GAS strains by assessment of IL-8 degradation both via ELISA (top panel) and Western blot (bottom panel). Error bars represent standard deviation, statistical analysis was carried out using a two-tailed *t*-test (ns = *p* > 0.05 ^**^*p* ≤ 0.01, ^***^*p* ≤ 0.001, ^****^*p* < 0.0001).

Figure S2 **Assessment of cell-surface host proteins by ELISA. (A)** Surface expression of the adhesion molecules ICAM and VCAM and of the endothelial cells integrity markers CD144 and trombomodulin (TM) on EA.hy926 cells after exposure to buffer only (control), rSpyCEP (10 μg/ml) or an unrelated protein (S1LG, 10 μg/ml) purified using the same method. The graph represents two pooled experiments and error bars represent the standard deviation.

Figure S3 **Scanning electron microscopy of GAS WT (A) and GAS Δ*cepA* (B)**. Mid-log phase bacterial culture was fixed on cover glass. Each strain observed with FE-SEM displayed the surface structure and both of them had similar surface quality. Scale bar represents 100 nm.
