# Supplementary figures and images for: The IL-8 protease SpyCEP is detrimental for Group A Streptococcus host-cells interaction and biofilm formation
Source: Front Microbiol. 2014 Jul 10;5:339. doi: 10.3389/fmicb.2014.00339 (PMC4090674; doi:10.3389/fmicb.2014.00339)

Figure S1

**A** SpyCEP antiserum binding

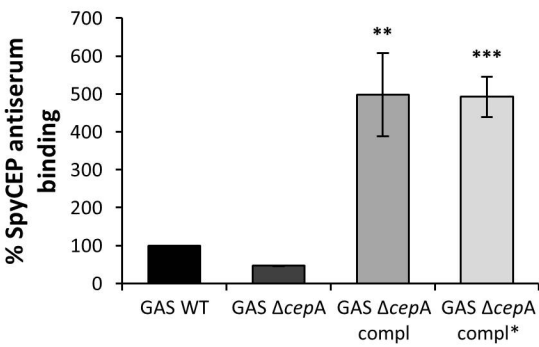

**B** IL-8 degradation: ELISA and western blot

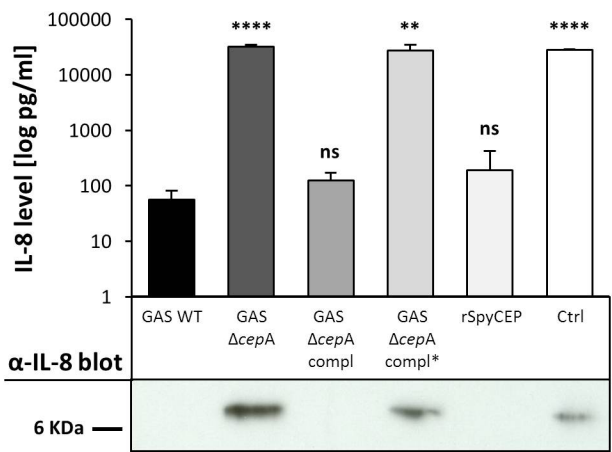

Supplement: Figure S1 — Expression and activity of SpyCEP*. (A) FACS analysis of anti-SpyCEP antiserum binding to various GAS strains' surface. (B) Quantification of SpyCEP activity in the supernatants of the various GAS strains by assessment of IL-8 degradation both via ELISA (top panel) and Western blot (bottom panel). Error bars represent standard deviation, statistical analysis was carried out using a two-tailed t-test (ns = p > 0.05 **p ≤ 0.01, ***p ≤ 0.001, ****p < 0.0001). [file DataSheet1.PDF]

Figure S2

A

Cell surface ELISA

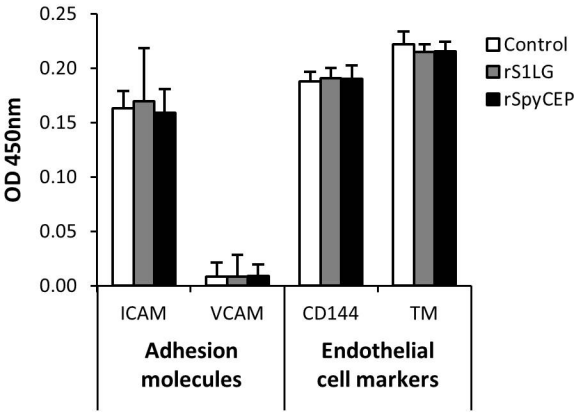

Supplement: Figure S2 — Assessment of cell-surface host proteins by ELISA. (A) Surface expression of the adhesion molecules ICAM and VCAM and of the endothelial cells integrity markers CD144 and trombomodulin (TM) on EA.hy926 cells after exposure to buffer only (control), rSpyCEP (10 μg/ml) or an unrelated protein (S1LG, 10 μg/ml) purified using the same method. The graph represents two pooled experiments and error bars represent the standard deviation. [file DataSheet2.PDF]

# Figure S3

**A: GAS WT**

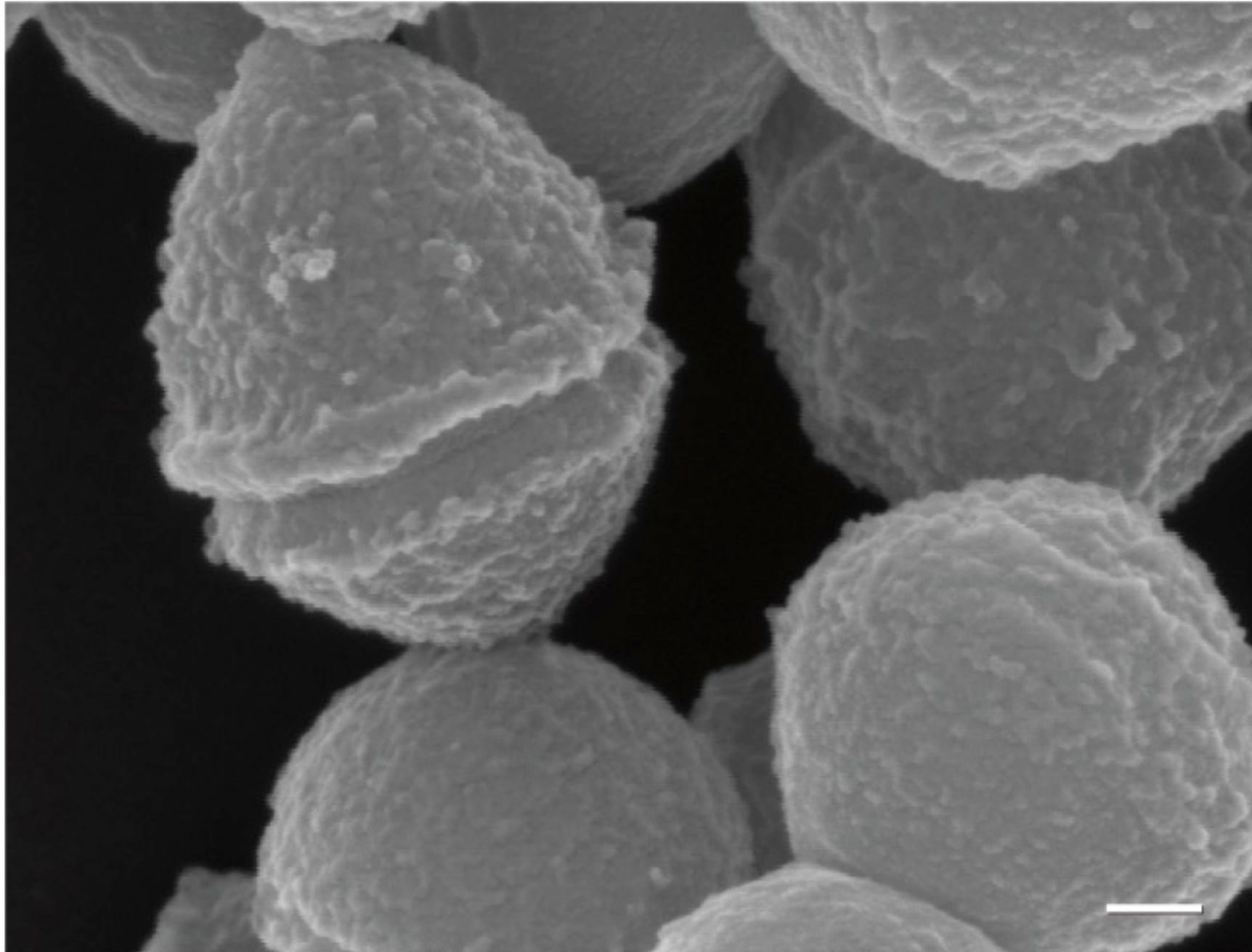

**B: GAS  $\Delta cepA$**

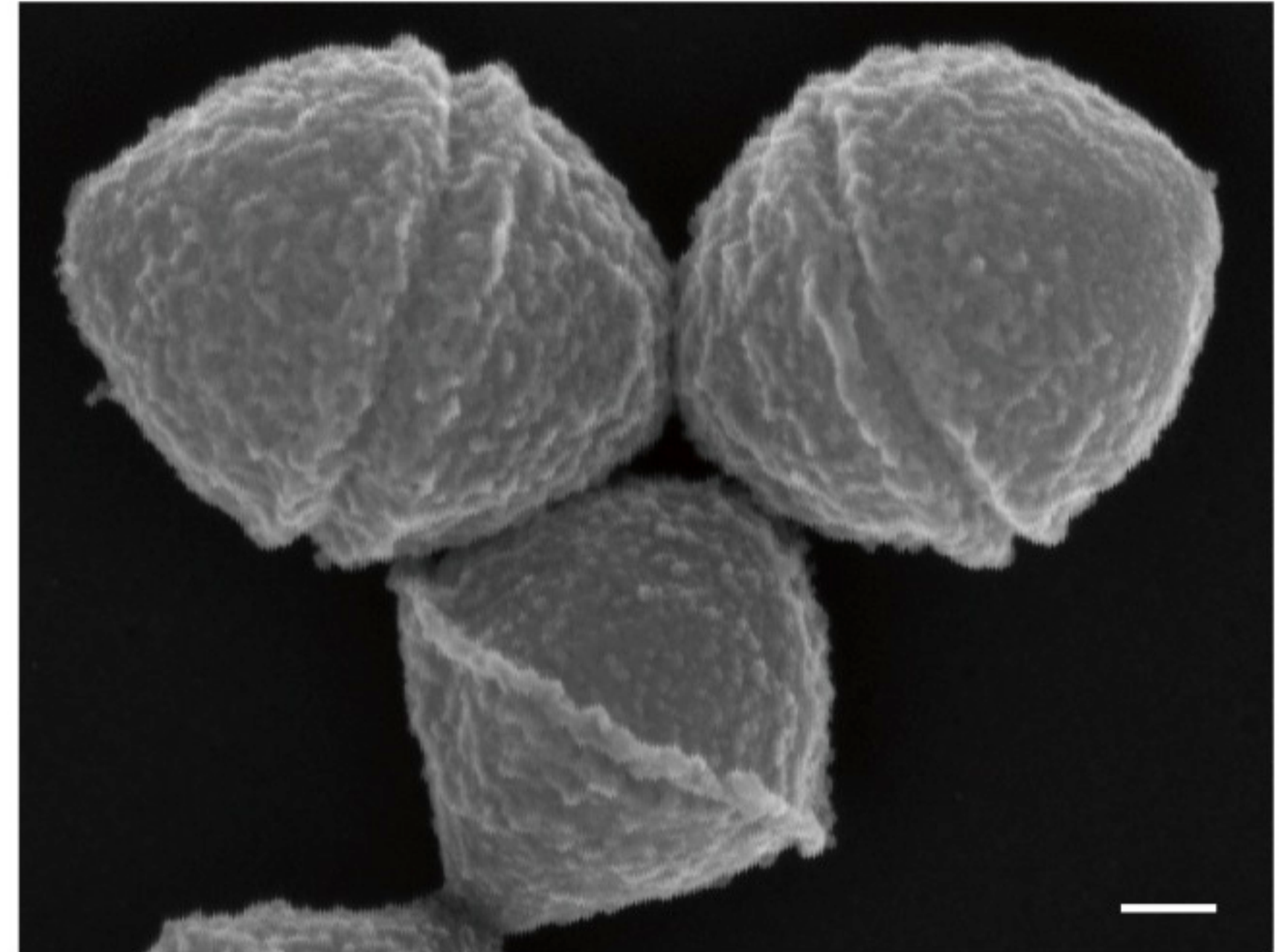

Supplement: Figure S3 — Scanning electron microscopy of GAS WT (A) and GAS ΔcepA (B). Mid-log phase bacterial culture was fixed on cover glass. Each strain observed with FE-SEM displayed the surface structure and both of them had similar surface quality. Scale bar represents 100 nm. [file DataSheet3.PDF]
